# COA-Cl (2-Cl-C.OXT-A) can promote coronary collateral development following acute myocardial infarction in mice

**DOI:** 10.1038/s41598-019-39222-1

**Published:** 2019-02-22

**Authors:** Toshiyuki Nishikido, Jun-ichi Oyama, Aya Shiraki, Ikuko Tsukamoto, Junsuke Igarashi, Koichi Node

**Affiliations:** 1 Department of Cardiovascular of Medicine, Saga University Hospital, Saga, Japan; 2 Department of Pharmaco-Bio-Informatics, Kagawa University, Kagawa, Japan; 3 Graduate School of Health Sciences, Morinomiya University of Medical Sciences, Osaka, Japan

## Abstract

2-Cl-C.OXT-A (COA-Cl) is a novel nucleic acid analogue that promotes tube-forming activity of human umbilical vein endothelial cells (HUVEC) through vascular endothelial growth factor (VEGF). The development of coronary collateral circulation is critical to rescue the ischemic myocardium and to prevent subsequent irreversible ischemic injury. We evaluated whether COA-Cl can promote angiogenesis in ischemic tissue, reduce infarct size and preserve cardiac contractility *in vivo*. Mice received COA-Cl or placebo daily for three days after myocardial infarction (MI) by coronary ligation. The degree of angiogenesis in ischemic myocardium was assessed by staining endothelial cells and vascular smooth muscle cells, and measuring infarct size/area-at-risk. In mice treated with COA-Cl, enhanced angiogenesis and smaller infarct size were recognized, even given a similar area at risk. We observed increases in the protein expression levels of VEGF and in the protein phosphorylation level of eNOS. In addition, the heart weight to body weight ratio and myocardial fibrosis in COA-Cl mice were decreased on Day 7. Administration of COA-Cl after MI promotes angiogenesis, which is associated with reduced infarct size and attenuated cardiac remodeling. This may help to prevent heart failure due to cardiac dysfunction after MI.

## Introduction

It has been recognized that collateral arteries tend to develop in ischemic vascular disease. Blood flow is increased either by angiogenesis, defined as the sprouting of new capillaries, or by the recruitment of pre-existing coronary collateral vessels. The development of collateral coronary circulation in the ischemic myocardium can salvage it from irreversible myocardial injury^1^. Therefore, it is known that coronary angiogenesis in the ischemic heart provides myocardial protective effects, potentially by reducing infarct size, subsequent myocardial dysfunction and incidence of arrhythmias. Angiogenesis is regulated by mechanical, chemical, and molecular factors^2^. Various peptide growth factors, including vascular endothelial growth factor (VEGF)^3^, hepatocyte growth factor^4^, and fibroblast growth factor (FGF)^5^ have been identified in cardiac angiogenesis due to myocardial ischemia^2,6^.

2-Cl-C.OXT-A (COA-Cl) was developed as a novel nucleic acid analogue that may possess the property of angiogenesis. Results of a previous study showed that the angiogenic activity of COA-Cl might confer clinical therapeutic value to COA-Cl as a novel angiogenic drug for ischemic stroke^7^. Our present study aimed to evaluate the angiogenic effect of COA-Cl after myocardial infarction (MI) *in vivo*, because expansion of collateral artery circulation in the ischemic myocardium leads to increased myocardial perfusion and eventual improvements in ventricular function.

## Results

### Reduced myocardial infarct size and remodeling in mice treated with COA-Cl

**Figure 1:**
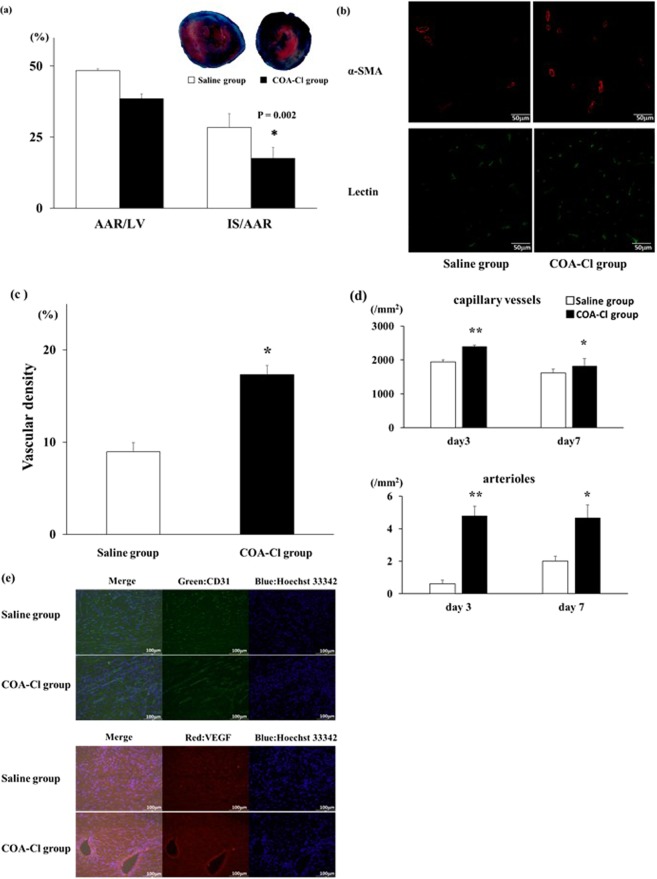
(**a**) Myocardial infarct size and area at risk on Day 3. The blue area is perfused tissue, the red and white area is the area at risk (AAR), and the white area is infarcted tissue (IS). The graphs show the IS as a percentage of left ventricular (LV) area (IS/LV), AAR as a percentage of LV area (AAR/LV), and IS as a percentage of AAR (IS/AAR). Saline group (n = 5) versus COA-Cl group (n = 5) *Significant difference by Student’s t-test at P < 0.05; (**b**) Immunofluorescent staining of α-smooth muscle actin (α-SMA) and lectin. Sections of hearts from the saline group (n = 5) versus the COA-Cl group (n = 5) 3 days after MI. Scale bar corresponds to 50 μm; (**c**) Vascular density in the border area was measured by quantitation of lectin staining. *Significant difference by Student’s t-test at P < 0.05. (**d**) The numbers of capillary vessels and arterioles (n = 4). *p < 0.05, **p < 0.01 vs saline group. (**e**) Immunofluorescent staining of CD31(upper panel) and VEGF (lower panel). Section of hearts from each group (n = 4) on day 7 after MI. Scale bar corresponds to 100 μm.

After MI, COA-Cl or saline was administered for three days. On Day 3 after MI, infarct size (IS) was reduced significantly in the group treated with COA-Cl (COA-Cl group) compared with the group treated with saline (saline group) (6.6 ± 0.6% versus 13.7 ± 1.6%, respectively; P < 0.01), and the area at risk (AAR), the ischemic area by LAD ligation, also tended to decrease in the COA-Cl group (38.6 ± 3.8% versus 48.4 ± 4.9%, respectively; P = 0.15). However, the IS/AAR ratio was smaller in the COA-Cl group than in the saline group (17.6 ± 1.6% versus 28.4 ± 2.2%, respectively; P < 0.01; Fig. 1a). We evaluated the effect of COA-Cl on cardiac dysfunction 7 days after MI with transthoracic echocardiography. The reduction of % fractional shortening (FS) and the dilation of the left ventricular dimension in the COA-Cl group were not as remarkable as those of the saline group. COA-Cl suppressed the dilation of the left ventricular dimension and decreased systolic function. Moreover, the heart weight (HW) to body weight (BW) ratio decreased significantly in the COA-Cl group (Table 1).

**Table 1:** The difference of echo parameter and cardiac/body weight between mice treated with COA-Cl and saline after myocardial infarction 7 day

Parameter	Sham (n = 6)	Saline group (n = 10)	COA-Cl group (n = 10)
BW, g	26.0 ± 1.0	25.7 ± 0.7	25.7 ± 0.8
HW, mg	156.3 ± 8.9	236.0 ± 0.14^#^	175.6 ± 13.9*
HW/BW, mg/g	6.02 ± 0.33	9.27 ± 0.65^#^	6.87 ± 0.60*
HR, bpm	451.7 ± 12.5	473.0 ± 16.2	491.4 ± 20.2
LVDd, mm	3.25 ± 0.14	3.94 ± 0.15^#^	3.54 ± 0.13
LVDs, mm	2.20 ± 0.11	3.12 ± 0.17^#^	2.46 ± 0.10*
IVS, mm	0.82 ± 0.03	0.71 ± 0.02^#^	0.76 ± 0.02
PW, mm	0.77 ± 0.06	0.74 ± 0.02	0.79 ± 0.02
FS, %	32.8 ± 1.6	21.3 ± 1.68^#^	29.0 ± 2.62*
EF, %	60.7 ± 2.7	47.4 ± 4.3^#^	63.7 ± 2.6 *

MI, myocardial infarction; BW, Body weight; HW, Heart weight; LVDd, diastolic dimension of left ventricle; LVDs, systolic dimension of left ventricle; IVS, thickness of interventricular septum; PW, thickness of posterior wall; FS, ratio of left ventricular fractional shortening; HR, heart rate; bpm, beats/min ^#^P < 0.05 vs.sham; *P < 0.05 vs. saline group.

### Preserved capillary density after myocardial infarction

We visualized the intramyocardial arteries and microvasculature 3 days after MI. Vasculardensity including capillary and arterioles in the heart was estimated with lectin specialized to bind to specific oligosaccharide side chains of vascular endothelial cells and α-smooth muscle actin antibody to bind to vascular smooth muscle cells. We quantified the blood vessel area stained by lectin in both the ischemic area and the border zone (Fig. 1b). The number of myocardial vessels including capillaries and arterioles in the COA-Cl group was significantly greater compared with that in the saline group (Fig. 1c–e), which proved to promote angiogenesis by COA-Cl in the ischemic area.

### Protein expression after treatment with COA-Cl

**Figure 2:**
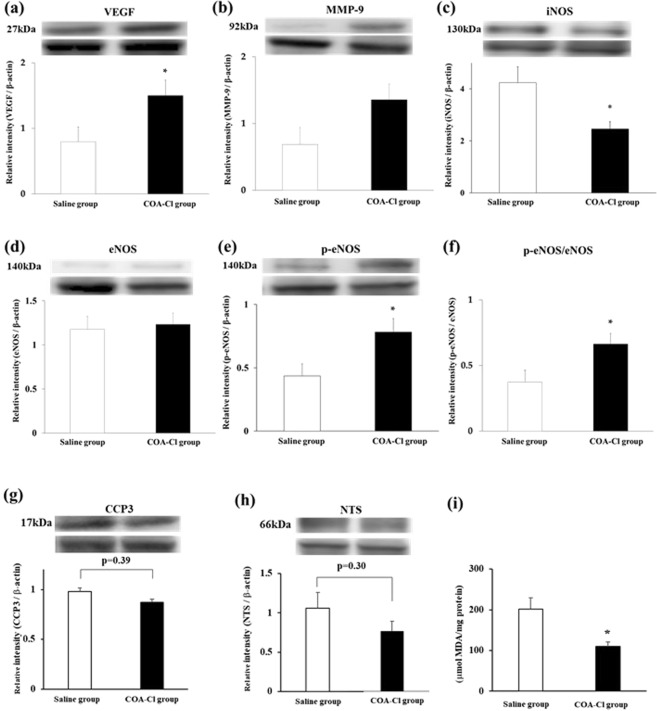
(**a**) Vascular endothelial growth factor (VEGF); (**b**) Matrix metalloproteinase (MMP)−9; **(c**) Inducible nitric oxide synthase (iNOS); (**d**) Endothelial nitric oxide synthase (eNOS); (**e)** phosphor-eNOS; (**f**) phosoho-eNOS/eNOS; (**g**) Cleaved caspase-3 (CCP3); (**h**) Nitrotyrosine (NTS) in heart measured by Western blot analysis. (**I**) Myocardial TBARS assay. Saline group (n = 8) versus COA-Cl group (n = 8). β-actin was used as a control (45 kDa: lower band above the graph). *Significant difference by Student’s t-test at P < 0.05.

**Figure 3:**
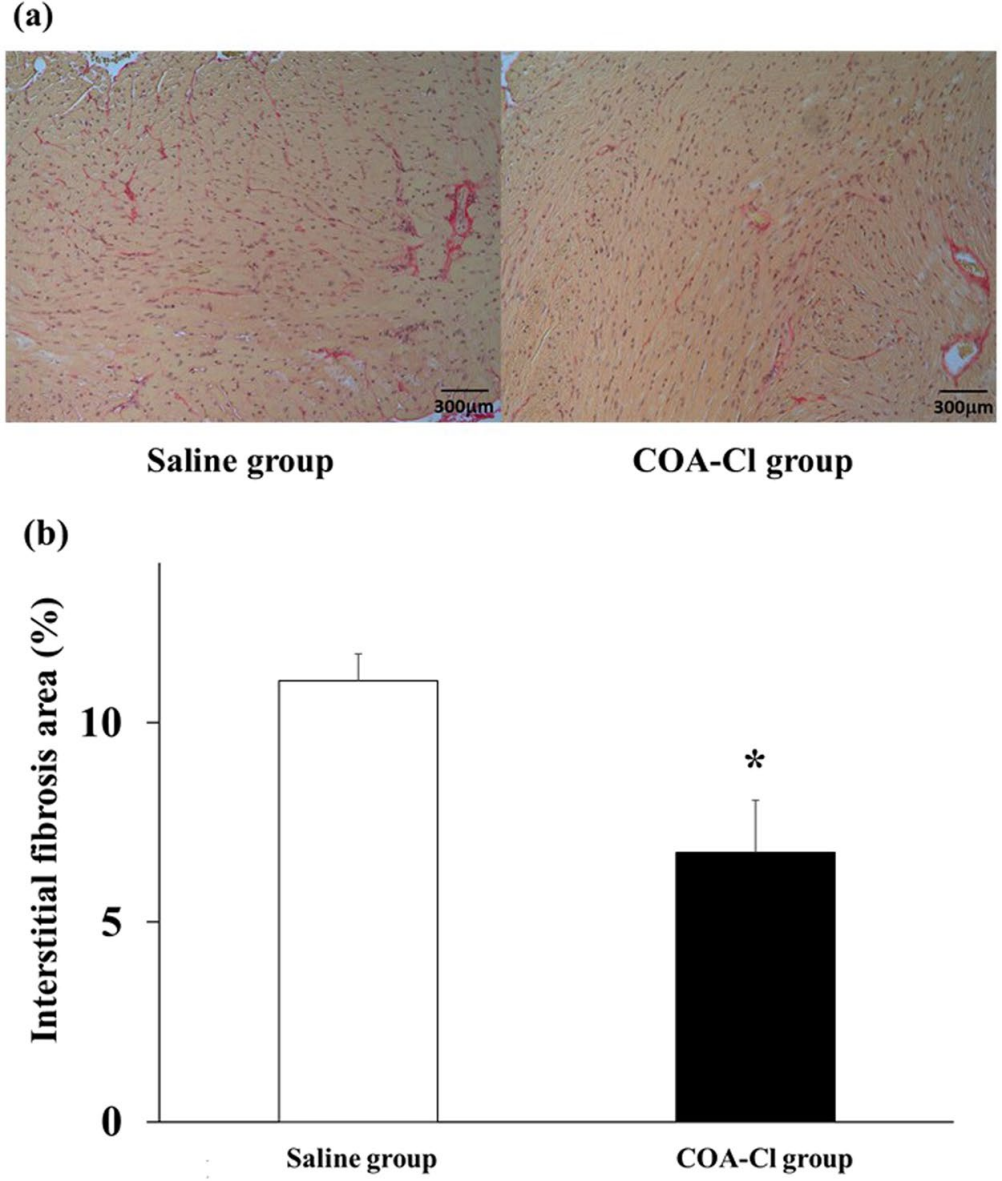
(**a**) Immunohistochemical staining of hearts in the saline group (n = 5) and the COA-Cl group (n = 5) on day 7. Both sections were stained by Sirius red stains. Scale bar corresponds to 300 μm; (**b**) fibrosis was measured by quantitation with Sirius red staining. *Significant difference by Student’s t-test at P < 0.05.

VEGF expression in the COA-Cl group was increased significantly compared to that in the saline group, as documented by Western blot analysis (P = 0.04; Fig. 2a). Furthermore, eNOS expression was not different between the saline group and the COA-Cl group. In contrast, the expression of phosphor-eNOS and phosphor-eNOS/eNOS was increased in the COA-Cl group (Fig. 2d–f; P = 0.78, 0.03, and 0.03, respectively). The degree of eNOS phosphorylation was higher in the COA-Cl group than in the saline group, although the amount of total eNOS protein expression was not. MMP-9 tended to be expressed more in the COA-Cl group (P = 0.07; Fig. 2b). Previous studies indicated that these factors also have a role in the progression of angiogenesis^8,9^. iNOS was induced by the inflammatory response after myocardial damage^10^, which was less in the COA-Cl group (P < 0.01; Fig. 2c). This finding suggests that collateral arteries in the ischemic region increased blood flow, which suppressed necrosis of myocardial tissue and the subsequent inflammatory response. Moreover, there was no significant difference in the expression of cleaved caspase-3 (CCP3) between the groups (P = 0.392; Fig. 2g), however, COA-Cl tended to reduce the expression of nitrotyrosine (NTS) by 28% (P = 0.302; Fig. 2h) and the level of TBARS was significantly reduced (P < 0.05; Fig. 2i). On Day 7 after MI, myocardial interstitial fibrosis was significantly less in the COA-Cl group than in the saline group. (6.8 ± 1.3 vs. 11.1 ± 0.7, respectively; P = 0.02; Fig. 3) These results indicated that COA-Cl reduced oxidative stress, and infarct area by enhancing blood vessel formation after myocardial infarction (MI), and prevented subsequent cardiac dysfunction.

## Discussion

In this study, we demonstrated that COA-Cl reduced infarct area and developed myocardial collateral vessels with activating angiogenic molecules such as VEGF, eNOS, and MMP-9 in murine MI model which leads to suppressing inappropriate cardiac remodeling. These results suggested that the administration of COA-Cl resulted in suppression of cardiac damage and subsequent cardiac remodeling due to strong angiogenic activation.

Clinically the development of collaterals in the ischemic myocardium is critical to reduce jeopatized ischemic tissue and prevent LV dysfunction resulting in heart failure., which is caused by angiogenic factors including hypoxia, inflammation, hemodynamics, and shear stress^2^. Therefore, along with the immediate implementation of coronary angioplasty to salvage the ischemic myocardium, it is desirable to establish a method of treatment aimed at protecting the ischemic myocardium by forming sufficient collateral circulation and attenuating the ischemic damage.

Previously we showed that COA-Cl induced a strong angiogenic response in cultured human umbilical vein endothelial cells (HUVEC) that are co-cultured with fibroblasts, as well as in chicken chorioallantoic membrane and rabbit corneal matrigel implant models^8^, and COA-Cl promoted the phosphorylation and activation of the mitogen-activated protein kinase (MAPK) cascade, which caused angiogenesis involving the G-protein coupled sphingosine 1-phosphate receptor 1 (S1P_1_), which can modulate angiogenesis, in the tube-forming activity of HUVECs^8,9^. As serum-borne lysophospholipid sphingosine 1-phosphate (S1P), endogenous ligand for S1P1, is produced in various cell types including vascular endothelial cells^10,11^, S1P-S1P_1_ pathway acts to regulate vascular maturation as a vascular intrinsic stabilization mechanism^12^, which converts extracellular stimulation by COA-Cl into intracellular signaling and then leads to the development of angiogenesis^9^.

COA-Cl also induces the expression and secretion of VEGF in the presence of normal human dermal fibroblasts, which is caused by the expression of a transcriptional coactivator peroxisome proliferator-activated receptor-γ coactivator (PGC)−1α in concert with ERRα^13^. VEGF has a high angiogenic potential that regulates angiogenesis and vasculogenesis, as well as vascular maintenance. In addition, it is the strongest inducer of vascular permeability due to efficient simultaneous nitric oxide (NO) and prostacyclin production^14,15^, which is the necessary step for angiogenesis by providing endothelial cell proliferation and migration^16^. Therefore, COA-Cl may not only prompt angiogenesis in the process of sprouting vascular endothelial cells initially by the induction of VEGF, but also stimulate mature endothelial tube formation gradually via the S1P-S1P_1_ pathway. We also confirmed that COA-Cl induces VEGF expression and secretion in fibroblasts from adjacent fibroblasts, which is promoted by expression not of HIF1α but of PGC-1α^17^. Moreover, COA-Cl may also cause a stronger angiogenic response by activating the S1P-S1P_1_ system in endothelial cells.

In this study, VEGF and eNOS were activated by COA-Cl after MI, but iNOS expression induced by inflammatory cytokines was decreased, despite the fact that VEGF induces eNOS and iNOS expression in vascular endothelial cells^18^. This might be because COA-Cl promoted angiogenesis due to the effect of VEGF and eNOS, and then enhanced blood perfusion that attenuated inflammation after MI, leading to declining iNOS activity. In post-infarct myocardium, angiogenic growth factors and inflammatory cytokines induce matrix metalloproteinase (MMP)^19^, which has a beneficial role in proper wound healing in early stages of cardiac remodeling, thus contributing to tissue replacement and scar formation^20^. In addition, it was shown that MMP-9 has a significant role in neovascularization through the proteolytic degradation of the basal lamina in the blood vessels and release of activated VEGF^21^. The results of the present study showed that MMP-9 expression was greater with COA-Cl, which might be caused by the degradation of the extracellular matrix with subsequent activation of major proangiogenic factors, such as VEGF in vasculogenesis.

Certainly, VEGF is one of the major angiogenic factors and is produced early in the angiogenic cascade and is associated significantly with the initial activation of endothelial cells^22^ to proliferate and result in sprouting in angiogenesis. However, the administration of VEGF for ischemic heart disease was not sufficient to induce angiogenesis in a clinical study^23^. Despite the fact that angiogenic factors, including VEGF, would have potential clinical therapeutic value, they are unstable and difficult to synthesize chemically and biologically, in addition to being expensive. On the other hand, COA-Cl is a Xeno-free novel adenosine-like nucleic acid analogue, and it is stable and easy to synthesize. Therefore, COA-Cl may have potential benefits as a novel treatment for increasing collateral circulation for ischemic diseases including coronary artery disease, stroke, and peripheral artery disease. However, there is a possibility to increase nutrient blood vessels for tumor etc. and to advance cancer extension, so further research including safety is required. Moreover, clinical studies of therapeutic angiogenesis with COA-Cl will be required to demonstrate long-term clinical benefits on the morbidity and mortality of ischemic disease as well as pharmaceutical toxicity. In addition, it is hard to deny other cardioprotective possibilities of COA-Cl through unknow mechanisms beyond neovascularization. The results of future basic and clinical researches on COA-Cl will be awaited.

In summary, COA-Cl promotes the development of collateral artery circulation after MI, which leads to reduced infarct size and cardiac remodeling. COA-Cl has potential as a novel therapeutic agent for ischemic heart disease; therefore, we expect further studies of the pharmacological effects of COA-Cl to improve clinical outcomes.

## Materials and Experimental Procedures

### Experimental animals and experimental design

C57 BL/6 mice purchased from Kyudo Co., Ltd. (Tosu, Japan) were housed in a specific pathogen-free environment. Healthy adult males were aged 12 to 16 weeks and weighed 20 to 25 grams. All experiments were performed in accordance with the “Position of the American Heart Association on Research Animal Use” and approved by the Institutional Animal Care and Use Committee at Saga University.

### Surgery to induce myocardial infarction

Mice were anesthetized with ketamine (100 ml/kg) + xylazine (10 mg/kg) and intubated for mechanical ventilation. After left thoracotomy to expose the heart, MI was achieved by ligation of the left anterior descending coronary artery with an 8-0 silk suture. After closure of the chest wall and extubation, the hearts were harvested 3 or 7 days later. Mice were randomly assigned to two groups: 12 mg/kg COA-Cl (COA-Cl group)^24^ or saline (saline group) were administered intraperitoneally right after MI for 3 days. We confirmed that there are no significant differences in heart weight and data about hemodynamics using COA-Cl among sham-operated mice.

### Measurement of infarct size

After internal carotid artery cannulation, 1% Evans blue dye was infused into the aorta and coronary artery to assess the area at risk. The hearts were sliced transversely into 2-mm thick sections and incubated with 1% triphenyltetrazolium chloride solution (TTC) (Sigma Aldrich Chemical, Buchs, Switzerland) at 37 °C for 15 minutes as previously described^25^. Each section was stained in a different color for the infarct area (IS), area-at-risk (AAR), and viable myocardium, and each color was measured with Image J software.

### Echocardiographic analysis

Echocardiographic analysis was performed with an echocardiographic machine with a 15-MHz transducer (Toshiba, Japan) at 7 days after MI, as described previously^26^. From 2-dimensional short-axis imaging with the M-mode, left ventricular end-diastolic diameter (LVEDD) and end-systolic diameters (LVESD) were measured, and fractional shortening was calculated as {(LVEDD-LVESD)/LVEDD × 100 (%FS) as described previously^26^. The left ventricular ejection fraction (LVEF) was calculated using the formula EF% = [(LVEDV − LVESV)/LVEDV] × 100; where LVEDV and LVESV are left ventricular end-diastolic volume and left ventricular end-systolic volume, respectively.

### Histological analysis

Samples were taken from the cardiac tissue around the infarct regions and fixed in 4% formalin for 24 hours, dehydrated in 70% ethyl alcohol, and embedded in paraffin before cryopexy. Left ventricular sections (5 µm) were cut and stained with hematoxylin-eosin. Immunofluorescence staining for neovascularization was visualized on a Zeiss confocal microscopy system, and then blood vessel densities were compared. New capillary blood vessels, including vascular endothelium and smooth muscle, were stained with α-smooth muscle actin (α-SMA) (A2547, 1:1000) (Sigma Chemical Co. Lt. Louis, MO) and Griffonia simplicifolia Lectin I- ISOLECTIN B4 (LECTIN) (FL-1201, 1:3000) (Vector Laboratories Ltd., Peterborough, UK). In addition, collagen density due to fibrosis was assessed with Sirius red staining. For each myocardial specimen, 10 independent fields from of 5 stained sections were photographed at 200x magnification. All areas were analyzed by quantitation with Image J software.

### Assay for protein expression

Heart sections were homogenized in cytoplasmic and nuclear extraction regents (Thermo scientific, Hudson, NH) containing protease inhibitors. Samples of myocardium lysate were resolved on SDS-PAGE according to a standard protocol. After protein transfer to membranes, the membranes were probed with primary antibodies followed by secondary antibodies conjugated to horseradish peroxidase, and immunoreactive bands were developed with ECL Plus Western Blotting Detection Regents (GE Healthcare, Buckinghamshire, UK). Enhanced chemiluminescence was detected with an LAS-3000 image analyzer (Fujifilm, Tokyo, Japan), and band density was quantified with Image J software. We used the following primary antibodies: inducible nitric oxide synthase (iNOS) (#6 10432, 1:1000) (BD Transduction Laboratories, Lexington, KY), endothelial nitric oxide synthase (eNOS) (#9586, 1:1000), Phospho-eNOS (Ser1177) (#9570, 1:1000), MMP-9 (#3852, 1:1000) (Cell Signaling, Danvers, MA), β-actin I-19 (sc-1616, 1:1000), VEGF (A-20) (sc-152, 1: 200) (Santa Cruz Biotechnology, INC., Santa Cruz, CA), cleaved caspase-3 (CCP3) and nitrotyrosine (N0409, 1:1000) (Sigma-Aldrich, St. Louis, MO). Myocardial TBARS was measured as previously described (Cayman Chemical, Ann Arbor, MI)^26^.

### Statistical analysis

Data are expressed as mean ± SEM. Multiple group comparison were analyzed using one-way ANOVA with Tukey post-hoc multiple comparison. Comparisons between groups were made with a two-sample t-test assuming equal variances. A P-value less than 0.05 was deemed statistically significant. All statistical analysis was performed with the SPSS software statistics package (version. 22; IBM SPSS, Chicago, IL).
